# Cerebral microbleed burden after aortic and cardiac surgery is associated with extracorporeal circulation exposure: Evidence from young patients with heritable connective tissue disorders

**DOI:** 10.1177/0271678X261499072

**Published:** 2026-10-20

**Authors:** Gianfranco Vornetti, Riccardo Regazzo, Valentina Barone, Valentina Belacchi, Giuliana La Rosa, Elisabetta Mariucci, Andrea Donti, Caterina Tonon, Raffaele Lodi, Luca Spinardi

**Affiliations:** 1 Programma Neuroimmagini Funzionali e Molecolari, IRCCS Istituto delle Scienze Neurologiche di Bologna, Bologna, Italy; 2 Dipartimento di Scienze Biomediche e Neuromotorie, Università di Bologna, Bologna, Italy; 3 Dipartimento di Scienze Mediche e Chirurgiche, Università di Bologna, Bologna, Italy; 4 UO Interaziendale Clinica Neurologica Metropolitana, IRCCS Istituto delle Scienze Neurologiche di Bologna, Bologna, Italy; 5 Azienda Sanitaria Provinciale di Catania, P.O. “Castiglione-Prestianni”, Bronte, Catania, Italy; 6 IRCCS Azienda Ospedaliero, Universitaria di Bologna, Bologna, Italy

**Keywords:** Aortic surgery, cerebral microbleeds, extracorporeal circulation, heritable connective tissue disease, susceptibility-weighted imaging (SWI)

## Abstract

**Background and purpose:**

Cerebral microbleeds (CMBs) occur after surgery involving extracorporeal circulation (ECC), though confounding risk factors may obscure this association. Patients with heritable connective tissue diseases (HCTDs) requiring early surgery provide an ideal model to investigate ECC-related CMBs. We aimed to assess CMB prevalence, distribution, and surgical correlates in a genetically confirmed HCTD population.

**Methods:**

We retrospectively analyzed 111 HCTD patients with brain magnetic resonance. CMBs number and location were correlated with clinical and surgical data, including procedure frequency, type, and, when available, ECC duration.

**Results:**

All 58 patients exposed to surgery under ECC exhibited CMBs, compared to only four surgery-naïve individuals. CMBs showed a predominantly lobar pattern. CMB burden was significantly higher in patients with multiple surgeries and longer ECC duration (*p* < 0.001), whereas valve type and surgical technique showed no association.

**Discussion:**

In our cohort, CMBs were identified in all individuals with ECC exposure. The absence of significant differences in CMB burden across surgical techniques weakens the hypothesis that CMBs originate solely from metallic microemboli. In young, normotensive patients with HCTDs, ECC exposure was uniformly associated with CMBs, with burden increasing with repeated procedures and ECC duration.

## Background and purpose of the study

Cerebral microbleeds (CMBs) are defined as small, round, or ovoid hypointensities smaller than 10 mm detected by magnetic resonance imaging (MRI) on T2*-weighted gradient-recalled echo (GRE) or susceptibility weighted imaging (SWI) sequences.^1^

CMBs are a hallmark of cerebral small vessel disease (SVD) and cerebral amyloid angiopathy (CAA).^2–5^ Recent studies have described the appearance of new lesions on SWI sequences in patients with a history of cardiac or aortic surgery involving extracorporeal circulation (ECC) or extracorporeal membrane oxygenation (ECMO), though their precise pathophysiological nature is not yet fully understood. Throughout this manuscript we use the term CMB in the established radiological sense; we emphasize, however, that the term is descriptive, and that the histopathological substrate of these lesions in the postoperative setting has not been demonstrated.

Initial reports in patients with mechanical heart valves (mHV) suggested these lesions were caused by metallic microemboli resulting from device wear and tear^6,7^; however, this hypothesis was later questioned when similar findings were observed in patients with modern mHVs, composed largely of pyrolytic carbon, and in those with biological heart valves (bHV).^8^ Moreover, CMBs have also been reported in patients undergoing ECC without valve replacement (e.g. coronary artery bypass grafting (CABG)) and in those receiving ECMO for acute respiratory failure.^9^

These observations, together with the reported correlation between lesion burden and ECC duration,^10–13^ have shifted attention toward ECC itself and its associated maneuvers, through mechanisms that may include contact activation of inflammation and coagulation, blood–brain barrier disruption, and gaseous embolism upon aortic declamping.^13–16^

Attribution is, however, difficult in the populations studied so far. Most series involve patients undergoing CABG or valve surgery, who are typically elderly and have multiple vascular risk factors, making it sometimes challenging to distinguish new ECC-related CMBs from those due to other etiologies, mainly SVD or CAA.^17^ Pediatric cohorts avoid this confounding and have shown lobar-predominant CMBs to be significantly more frequent after ECC than in matched controls, but are limited by small samples.^18,19^

In this context, patients with heritable connective tissue diseases (HCTDs) represent a particularly valuable population for investigating ECC-related SWI lesions. The causative mutations impair the structural integrity of the tunica media of large elastic arteries while sparing the microcirculation.^20,21^ Affected individuals undergo brain MRI at a young age to screen for intracranial arterial pathology^22,23^ and frequently require cardiac or aortic surgery^24,25^ before any risk factors for SVD or CAA develop. In this study, we therefore assessed whether ECC exposure is associated with the burden of SWI lesions in a cohort of young patients with genetically confirmed Marfan syndrome (MFS) or Loeys–Dietz syndrome (LDS), a model population free of microvascular comorbidity confounders.

## Materials and methods

### Study population

We retrospectively collected data on all patients (*n* = 260) with heritable connective tissue disease (HCTD) followed at our institution (Hub Center for Heritable Connective Tissue Diseases, University of Bologna, Italy). Patients were included if they met the following criteria: (1) a genetically confirmed diagnosis of MFS or LDS; (2) availability of at least one brain MRI scan; and (3) inclusion of SWI sequence in the MRI protocol. Exclusion criteria were a history of cerebrovascular disease or neurological deficits upon clinical examination. Of the initial 260 patients, 149 were excluded: 95 had not undergone brain MRI, and 54 had brain MRI performed with susceptibility-sensitive sequences other than SWI (i.e. T2* GRE). The latter were primarily identified in older examinations performed before the implementation of standardized imaging protocols including SWI. The final study population consisted of 111 patients with genetically confirmed MFS (*n* = 81, 73%) or LDS (*n* = 30, 27%). All patients underwent at least one MRI scan with SWI sequence between December 2018 and July 2024. When multiple MRI studies were available, the scan performed closest to the time of the last cardiac or aortic surgery was selected for analysis. Demographic and clinical characteristics of the patients were collected, including age, sex, type of genetic mutation, and presence of cardiovascular comorbidities, as well as the number and type of cardiac or aortic surgical interventions and endovascular/interventional procedures. When heart valve replacement was performed, classification was made according to the prosthesis type: mHV or bHV. When available, data on ECC duration was also recorded. For patients with a history of multiple ECC procedures, the durations of each procedure were summed to obtain a total ECC exposure.

### MRI acquisition and image analysis

An MRI scan of the brain was performed using a 1.5 T scanner (Ingenia; Philips Healthcare) with a 20-channel phased array head coil. The imaging protocol included the following sequences: sagittal T1-weighted GRE, axial fluid-attenuated inversion recovery (FLAIR) T2-weighted, coronal T2-weighted fast spin echo (FSE), diffusion-weighted imaging (DWI), 3D time-of-flight (TOF) angiography, and SWI. CMBs were identified on SWI images, acquired with the following parameters: field of view (FOV): 210 × 178 mm, matrix: 232 × 168 mm; resolution: 0.9 × 1.06 × 1.0 mm FOV; section thickness: 2 mm; gap: 1 mm; number of slices: 144; echo time (TE): 12 ms; repetition time (TR): 52 ms; and flip angle: 20°. The acquisition time for the SWI sequence was 4′23″. All MRI scans were independently reviewed by two blinded neuroradiologists (GV and LS) with 8 and 20 years of experience, respectively; disagreements in SWI lesions count were resolved by consensus. For each patient, the number of CMBs—defined as nodular hypointensities smaller than 10 mm on SWI sequences, as described in previous studies^26^—and their anatomical distribution (cerebral lobes, basal ganglia, thalami, brainstem, and cerebellum) were recorded. To differentiate paramagnetic hemosiderin deposits from diamagnetic substances, each lesion was cross-referenced with the corresponding SWI phase maps. To assess the presence of underlying SVD, the total SVD score (range 0–4) was calculated for each patient by assigning one point for each of the following: presence of lacunes, presence of CMBs, moderate-to-severe basal ganglia perivascular spaces, and confluent white matter hyperintensities (periventricular Fazekas score 3 or deep Fazekas score 2–3), as described by Staals et al.^27^ Because the CMB component of the score overlaps with the SWI lesions under investigation, a modified SVD score (range 0–3) was also computed by omitting the CMB item. The study was conducted in accordance with the Declaration of Helsinki. The study protocol was approved by the Area Vasta Emilia Centro Ethics Committee (CE-AVEC; reference no. EM624-2019_246/2016/O/Oss/AOUBo), and informed consent was obtained from all participants. The study conforms to the Strengthening the Reporting of Observational Studies in Epidemiology (STROBE) reporting guidelines.

### Statistical analysis

Continuous variables were described as mean ± standard deviation (SD), and categorical variables as absolute and relative (%) frequencies. The Shapiro–Wilk test was used to assess the normality of continuous variables. Because most distributions deviated from normality, nonparametric tests (Mann–Whitney *U* test or Kruskal–Wallis test) were applied for group comparisons, and the χ^2^ or Fisher exact test was used for categorical data. The number of SWI lesions was analyzed as a count outcome. Because the variance of SWI lesion counts largely exceeded the mean (indicating overdispersion), negative binomial regression models were used to estimate incidence rate ratios (IRRs) with 95% confidence intervals (CIs). Two main models were constructed. In the first, limited to patients with available ECC duration data, SWI lesion count was modeled as a function of ECC duration (per 10-min increase), surgical procedure type (Bentall with mechanical valve vs other aortic root surgeries), connective tissue disorder (MFS vs LDS), age, and sex. In the second, including the entire cohort, the number of previous surgeries replaced ECC duration as the primary predictor, with the same covariates. Multivariable results were reported as adjusted IRRs with 95% CIs. All statistical analyses were performed using R (version 4.3.0; R Foundation for Statistical Computing, Vienna, Austria). A two-sided *p* < 0.05 was considered statistically significant.

## Results

### Patient population and SWI lesions distribution

Among the 111 patients included in this study, 48 (43%) were female, and the mean age at the time of MRI was 37.9 ± 15.3 years. The mean interval between the most recent ECC-related operation and MRI was 4.8 ± 3.7 years. This interval was not associated with SWI lesion burden (*p* = 0.920). No patients had hypertension, dyslipidemia, or diabetes mellitus. Three patients (2.7%) were current smokers and 10 (9.0%) were past smokers. Fifty-three patients (47.7%) had no history of cardiac or aortic surgery, whereas 58 (52.3%) had undergone procedures involving ECC. Most of these patients (*n* = 41, 36.9%) had a single surgical procedure, while 17 (15.3%) had multiple surgeries. Nine patients (8.1%) underwent emergency surgery for aortic dissection. Elective surgical procedures were scheduled following multidisciplinary evaluation, in accordance with guideline recommendations. The most common operation was aortic root replacement, performed using either the David technique (*n* = 32, 28.8%) or the Bentall procedure (*n* = 22, 19.8%). Aortic valve replacement was performed with a bHV in four patients (3.6%) and with a mHV in 18 patients (16.0%). Additional interventions included thoracic aortic replacement with the frozen elephant trunk (FET) technique (*n* = 7, 6.3%), mitral valve replacement (*n* = 10, 9.0%), and heart transplantation (*n* = 2, 1.8%). Sixteen patients (14.4%) had undergone at least one vascular or neurovascular procedure not involving ECC: 12 of 58 patients (20.7%) with previous ECC exposure and four of 53 patients (7.5%) without ECC exposure. Thirteen patients had undergone one or more endovascular/interventional procedures, five had undergone one or more open vascular operations, and two had undergone both. The main procedures included thoracic descending or thoracoabdominal aortic endografting; open thoracoabdominal, abdominal aortic, or aortoiliac reconstruction; radial or popliteal artery aneurysm repair; intracranial aneurysm coiling and treatment of a dural arteriovenous fistula.

No significant differences were observed in demographic or surgical variables between patients with MFS and those with LDS. The demographic and clinical characteristics of the study population are summarized in Table 1. On SWI sequences, at least one CMB was detected in 62 patients (55.9%), including 58 with prior ECC exposure and four without. The total SVD score was one in the 62 patients with at least one SWI lesion and zero in the remaining 49; the modified score excluding the CMB item was zero in all patients, indicating no MRI evidence of underlying SVD. The mean number of SWI lesions was 10.5 ± 15.1. SWI lesions were predominantly located at the cortical–subcortical junction of the cerebral convexities, most frequently in the frontal lobe (25.2%), followed by the parietal (20%), temporal (16.7%), and occipital (9.8%) lobes, demonstrating a predominantly lobar distribution. However, deep SWI lesions involving the basal ganglia (4.5%), brainstem (3.5%), and thalami (2.7%) were also observed. Cerebellar SWI lesions were present in 15% of patients. A representative example of SWI lesion burden in patients with and without surgical interventions is illustrated in Figure 1.

**Table 1.** Demographic and surgical characteristics of the study population.

Variable	Overall (*n* = 111)	MFS (*n* = 81)	LDS (*n* = 30)	*p* Value
Sex (female)	48 (43.2%)	33 (40.7%)	15 (50.0%)	0.397
Age (years)	37.9 ± 15.3	37.8 ± 14.3	38.3 ± 18.1	0.877
History of cardiac or aortic surgery with ECC	58 (52.3%)	40 (49.4%)	18 (60.0%)	0.394
Aortic root replacement—Bentall technique	22 (19.8%)	15 (18.5%)	7 (23.3%)	0.597
Aortic valve replacement—bHV	4 (3.6%)	2 (2.5%)	2 (6.7%)	0.295
Aortic valve replacement—mHV	18 (16.2%)	13 (16.0%)	5 (16.7%)	1.000
Aortic root replacement—David technique	32 (28.8%)	22 (27.2%)	10 (33.3%)	0.638
Thoracic aorta replacement—FET	7 (6.3%)	5 (6.2%)	2 (6.7%)	1.000
Mitral valve replacement	10 (9.0%)	8 (9.9%)	2 (6.7%)	0.726
Heart transplantation	2 (1.8%)	1 (1.2%)	1 (3.3%)	0.469
Total SVD score				
0	49 (44.1%)	38 (46.9%)	11 (36.7%)	0.393
1	62 (55.9%)	43 (53.1%)	19 (63.3%)	
Modified SVD score (excluding CMBs)				
0	111 (100%)	81 (100%)	30 (100%)	1.000

ECC: extracorporeal circulation; FET: frozen elephant trunk; mHV: mechanical heart valve; bHV: biological heart valve; CMBs: cerebral microbleeds.

Data are presented as *n* (%) for categorical variables and as mean ± standard deviation for continuous variables.

**Figure 1.**
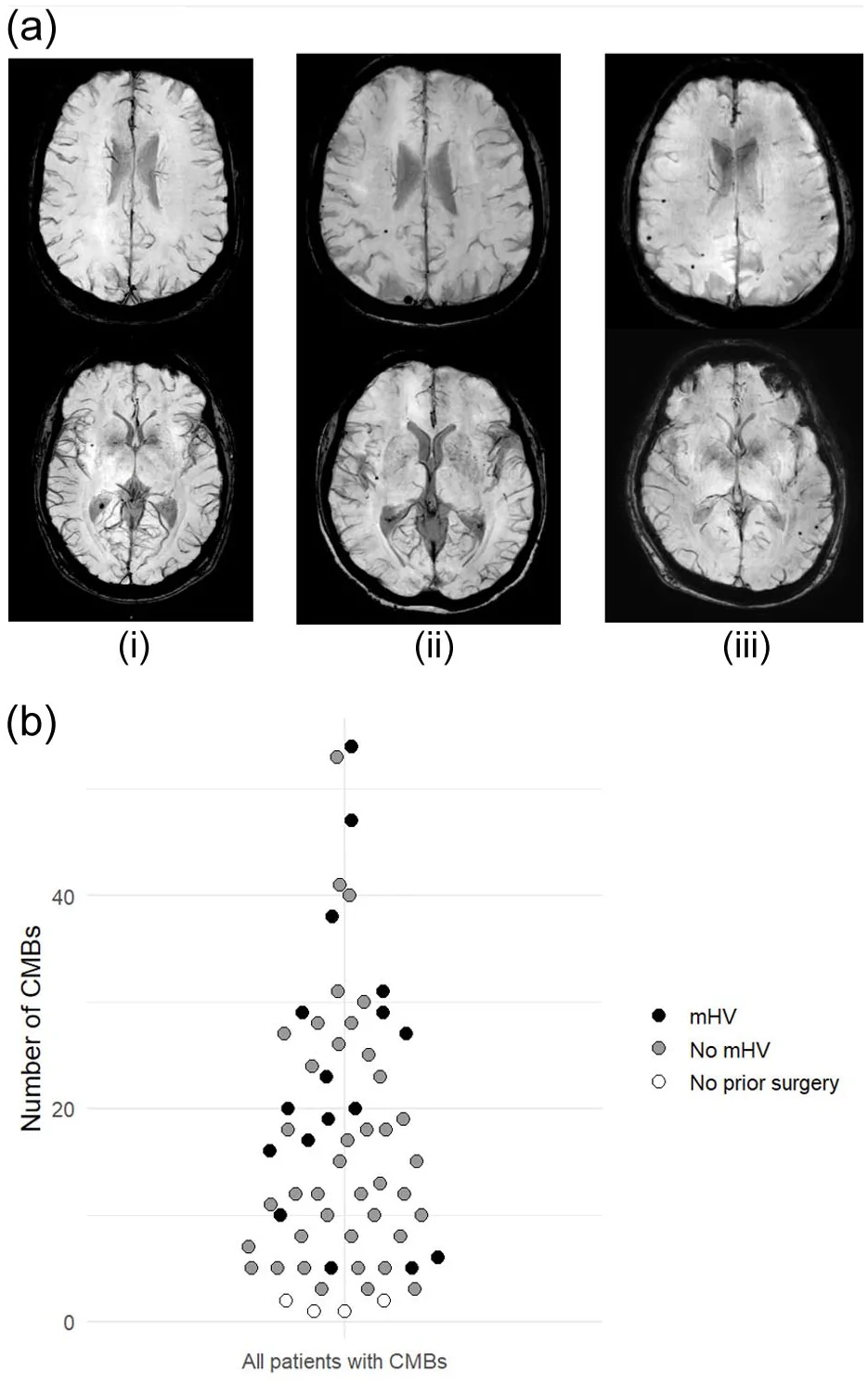
(a) Axial SWI sequences at the cella media of the lateral ventricle (top row) and basal ganglia (bottom row) illustrating the increasing CMB burden associated with surgical interventions involving ECC in three patients: (i) 39-year-old female, no surgery, CMB burden = 1, (ii) 62-year-old female, Bentall procedure with mHV, CMB burden = 20, and (iii) 52-year-old male, David procedure, CMB burden = 26 and (b) dot plot of CMBs per patient categorized by surgical history. Each dot corresponds to one patient with ⩾1 CMB. One outlier (CMB burden = 93, mHV) was omitted to improve readability. CMBs: cerebral microbleeds; mHV: mechanical heart valve.

### Association between cardiac or aortic surgery and SWI lesion burden

SWI lesions were significantly more frequent in patients who had undergone cardiac or aortic surgery (*p* < 0.001). Among those without a history of ECC, only four patients demonstrated SWI lesions (7.5%), with a maximum of two lesions. In contrast, all patients with previous surgery under ECC had SWI lesions, ranging from 3 to 93. Considering the entire patient cohort, the number of SWI lesions increased significantly with the number of surgical interventions under ECC (*p* < 0.001; Figure 2). Univariate analysis revealed significant association between SWI lesion burden, number of surgeries, mHV replacement, and older age. However, in the multivariate model, only the number of surgical procedures under ECC remained an independent predictor of SWI lesion count (Table 2). Data on ECC duration were available for 37 patients (63.8%), with a mean ECC time of 190.6 ± 99.3 min. In this subset, ECC duration showed a positive correlation with the number of SWI lesions, and was the only variable significantly associated with SWI lesion burden in both univariate and multivariate analysis (*p* < 0.001; Table 3). A representative example of SWI lesion burden in patients with varying ECC times is shown in Figure 3.

**Figure 2.**
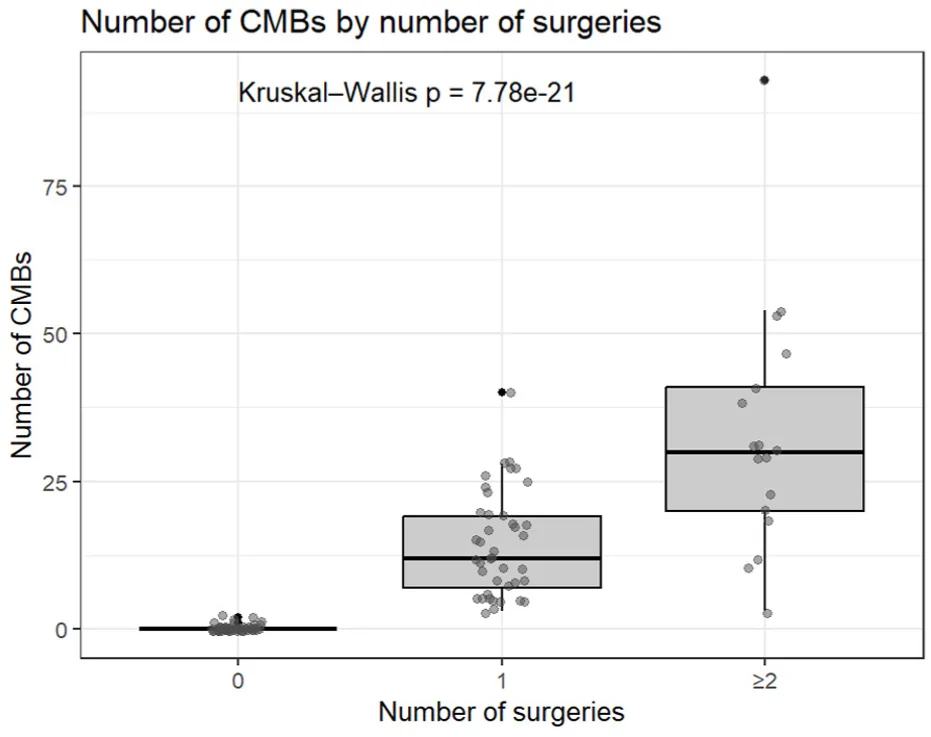
Distribution of CMB burden according to the number of prior cardiac surgeries. CMBs: cerebral microbleeds.

**Table 2.** Negative binomial regression analyses assessing the association between the number of interventions under ECC and the number of CMBs in the entire patient cohort (*n* = 111).

Variable	Univariate IRR (95% CI)	*p*	Multivariate IRR (95% CI)	*p*
Number of interventions under ECC	9.77 (5.34–17.85)	<0.001	9.29 (5.06–17.08)	<0.001
Procedure: Bentall + mHV (vs others)	3.75 (2.34–6.00)	<0.001	0.79 (0.43–1.45)	0.446
Condition: MFS (vs LDS)	0.71 (0.41–1.21)	0.207	0.66 (0.42–1.04)	0.074
Age (years)	1.04 (1.02–1.06)	<0.001	1.01 (0.99–1.03)	0.205
Sex: Male (vs Female)	1.38 (0.81–2.35)	0.239	1.08 (0.71–1.64)	0.731

ECC: extracorporeal circulation; mHV: mechanical heart valve; IRR: incidence rate ratio.

**Table 3.** Negative binomial regression analyses assessing the association between ECC duration and the number of CMBs among patients with available data on ECC duration (*n* = 37).

Variable	Univariate IRR (95% CI)	*p*	Multivariate IRR (95% CI)	*p*
ECC duration (+10 min)	1.04 (1.02–1.05)	<0.001	1.04 (1.02–1.05)	<0.001
Procedure: Bentall + mHV (vs others)	1.51 (0.89–2.57)	0.130	1.21 (0.87–1.69)	0.250
Condition: MFS (vs LDS)	0.77 (0.47–1.27)	0.307	0.87 (0.59–1.28)	0.478
Age (years)	1.00 (0.99–1.02)	0.621	1.01 (1.00–1.02)	0.146
Sex: Male (vs Female)	0.93 (0.59–1.47)	0.744	0.85 (0.60–1.21)	0.378

ECC: extracorporeal circulation; mHV: mechanical heart valve; IRR: incidence rate ratio.

**Figure 3.**
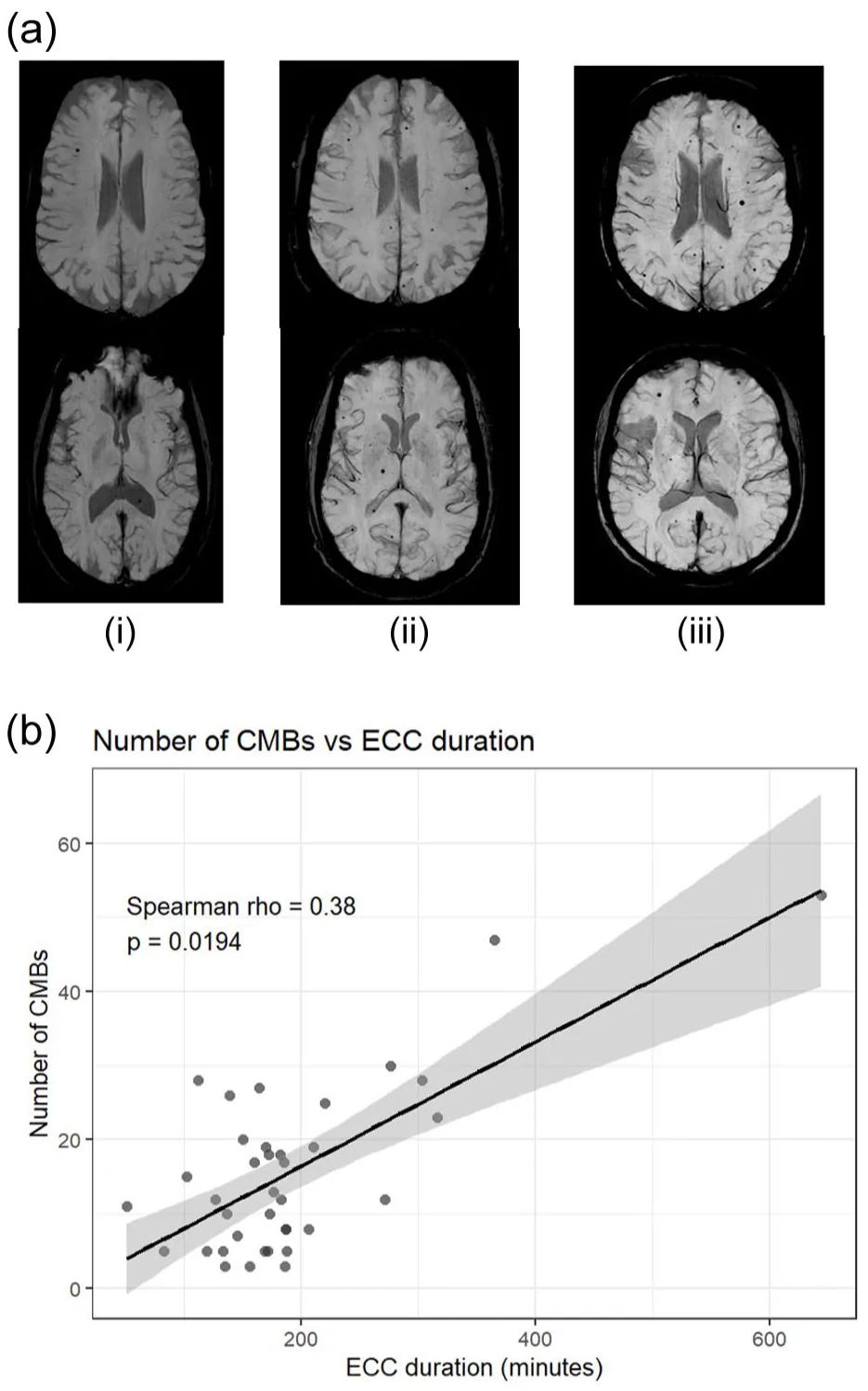
(a) Representative SWI images demonstrating CMB burden in three patients with different ECC durations. Top row: axial slices at the level of the cella media of the lateral ventricles and bottom row: axial slices at the level of the basal ganglia: (i) 22-year-old female with 51 min ECC, showing minimal CMB burden, (ii) 40-year-old female with 365 min ECC, indicating a moderate CMB burden, and (iii) 28-year-old male with 644 min ECC, displaying numerous CMBs and (b) correlation between CMB burden and ECC duration. CMBs: cerebral microbleeds; ECC: extracorporeal circulation.

The nine patients who underwent emergency surgery for aortic dissection had a higher SWI lesion count than those without (31.4 ± 15.4 vs 18.0 ± SD 15.3, *p* = 0.011). This difference did not persist after adjusting for the number of procedures performed under ECC (*p* = 0.63), which was higher in the dissection group (2.0 ± 0.9 vs 1.3 ± 0.5 procedures, *p* = 0.011).

The unadjusted SWI lesion count was higher in patients who underwent vascular/neurovascular procedures not involving ECC than in those without such exposure (22.5 ± 18.4 vs 8.4 ± 13.6, *p* < 0.001). However, after adjustment for the number of previous ECC procedures, non-ECC vascular/neurovascular exposure was not independently associated with SWI lesion burden (*p* = 0.880).

## Discussion

In our cohort, CMBs were observed in all patients with a history of cardiac or aortic surgery involving ECC, whereas the prevalence among surgery-naïve patients was 7.5%, consistent with estimates reported in the general population.^28^ Previous studies were either conducted on small pediatric cohorts^19^ or on older patients with multiple risk factors for SVD and CAA.^10^ While HCTDs lead to structural fragility of the tunica media in the great arteries, they have not been found to affect the cerebral microvasculature. Indeed, our cohort lacked the principal microvascular risk factors, including hypertension, dyslipidemia and diabetes mellitus, and exhibited a low prevalence of smoking, showing no clinical or radiological evidence of SVD or CAA.

Previous studies have identified a significantly higher CMB burden in valve replacement procedures compared to CABG.^10,17^ In our study, neither surgical technique nor valve type was significantly associated with CMB burden.

Although unadjusted analyses showed a higher SWI lesion burden both in patients who had undergone emergency surgery for aortic dissection and in those exposed to non-ECC vascular or neurovascular procedures, neither association remained statistically significant after adjustment for the number of previous ECC procedures. These findings suggest that the unadjusted differences may reflect the clustering of these exposures among patients with more complex vascular disease and a greater number of previous ECC procedures.

Although patients with mHVs showed a higher SWI lesion count—a finding that could be attributed either to the formation of metallic microemboli or to the requirement for lifelong anticoagulation—this difference lost significance in multivariate analysis. The observed increase in SWI lesion burden in valve replacement procedures may be explained by the longer ECC duration typically required, rather than by the valve prostheses themselves. To our knowledge, this is the first study to demonstrate a cumulative effect of repeated ECC exposures on SWI lesion. Patients who had undergone multiple surgeries with ECC exhibited substantially higher SWI lesion counts. Several mechanisms could contribute, including repeated microembolization, perioperative anticoagulation or coagulation disturbances, hemodynamic fluctuations and cerebral hypoperfusion, hemodilution or anemia, and inflammatory effects on blood–brain barrier integrity. Notably, in the subgroup of patients with available intraoperative records, our study also confirms a significant correlation between the duration of ECC and SWI lesion burden, thereby reinforcing the role of ECC in their pathogenesis. Specifically, we observed that each 10-min increase in ECC duration was associated with a 4% rise in the number of SWI lesions, highlighting the impact of procedural length on microvascular brain injury.

Our findings should therefore be interpreted as demonstrating an association between SWI lesion burden and cumulative ECC-related operative exposure, rather than identifying a single causal mechanism. Nevertheless, the marked difference in SWI lesion prevalence between ECC-exposed and unexposed participants, together with the associations of SWI lesion burden with ECC duration and the number of ECC procedures, supports a contribution from the ECC-related perioperative setting.

The higher prevalence of CMB in our cohort may reflect the significantly longer ECC duration in our population, with a mean of 190.6 ± 99.3 min compared to 90 ± 49 min in the study by Patel et al.,^10^ which evaluated patients undergoing CABG or valve replacement. This likely reflects the complexity of aortic root and arch procedures in HCTD.

Overall, our findings highlight the importance of considering ECC-related SWI lesions in the differential diagnosis when they are observed on MRI in patients with a history of cardiac or aortic surgery. Among the 53 patients without ECC exposure, four had SWI lesions on MRI. Of these, two patients presented with a single lesion, while two had two lesions each. The latter had both a history of prior interventional angiography: one for an intracranial aneurysm coiling and one for dural arteriovenous fistula repair, representing plausible non-ECC-related iatrogenic causes of CMBs.^29^ Nevertheless, our estimated prevalence in patients with no ECC exposure is similar to that observed in the general population.^28^

ECC-related CMBs were predominantly distributed along the cerebral convexities, with the frontal lobe most frequently involved, as also reported by Patel et al.^10^ The reason for the predominantly lobar distribution remains uncertain. One possible explanation is that gaseous or particulate microemboli generated during ECC or aortic manipulation may lodge within and injure distal cortical and subcortical microvessels. Perioperative hemodynamic disturbances, anticoagulation, and inflammation may also contribute.

Given the lobar distribution of these lesions, clinicians should be aware of their potential to act as confounders for CAA, since this can influence decisions regarding anticoagulation therapy and impact overall patient management, as described by De Sciscio et al.^17^ However, the Boston criteria were developed and validated for patients aged 50 years or older presenting with a potential CAA-related clinical syndrome, such as spontaneous lobar intracerebral hemorrhage, transient focal neurological episodes, or cognitive decline. They have not been validated as a screening instrument in asymptomatic individuals undergoing MRI for unrelated indications.^30^

Our cohort’s characteristics—namely, relatively young age, absence of hypertension or other primary microvascular risk factors, and lack of other common CMB etiologies—enhance the validity of our observations by minimizing potential bias from age-related or microvascular comorbidities that may have influenced previous studies. While pediatric series may reduce such biases, they are often limited by small sample sizes. In contrast, our population, with 111 patients, represents one of the larger cohorts documented in the literature.

Our study has several limitations. First, preoperative MRIs were unavailable for most patients, preventing the assessment of new SWI lesions relative to baseline. Second, detailed intraoperative data—including ECC duration and specific procedural parameters such as aortic cross-clamp time, use of deep hypothermic circulatory arrest, and transfusion requirements—were incomplete and available for only a subgroup of patients. Furthermore, due to the lack of a standardized follow-up protocol, the interval between surgery and MRI was heterogeneous and such variability may have introduced a potential bias in the assessment of the final SWI lesion burden. Additionally, data regarding non-cardiovascular and non-neurovascular operations, including orthopedic, ophthalmologic, and nonvascular abdominal surgery, were unavailable; therefore, residual confounding from these exposures cannot be entirely excluded, although routine procedures of these types have not been established as determinants of CMB burden. Finally, due to the cross-sectional and retrospective design of this study, we were unable to assess the long-term clinical consequences of ECC-related SWI lesions, such as possible neurocognitive or neurological effects.

Our findings demonstrate that ECC-related SWI lesions are frequent and characteristic MRI findings in young patients with HCTD undergoing cardiac or aortic surgery. Repeated exposures and ECC duration appear to be associated with greater SWI lesion burden, supporting a contribution from the ECC-related perioperative setting to microvascular injury. Definitive resolution of the nature of these lesions will require neuropathological correlation. Further studies are needed to assess their long-term neurological impact as well as to explore perioperative strategies—particularly efforts to minimize ECC duration—that may reduce the occurrence of these lesions.
